# Cystic lymphangioma of hepato-gastric ligament: a rare case of neoplasia in adults

**DOI:** 10.1186/1471-2318-11-S1-A22

**Published:** 2011-08-24

**Authors:** L Grimaldi, S Reggio

**Affiliations:** 1 Centro di Riferimento Regionale Tumori Rari (C.R.T.R.) – A.O.U. “Federico II” Napoli, Italy; 2 Assistente in Formazione in Chirurgia Generale, A.O.U. “Federico II” Napoli, Italy

## Background

The cystic lymphangiomas (LC) are polymorphous tumors of undecided origin, [1], observed mainly in children and more often localized in the cervical and axillary areas. [2]. We report the case of an adult who suffers from cystic lymphangioma of the peritoneum already with a history of surgery for the same type of cancer of the left cheek.

## Materials and methods

FE, a man of 71 years, comes to our attention in July 2009 complaining about one month from the onset of dyspeptic disorders and a sense of weight reported in epigastrium. In his history, in addition to this, the patient reported the removal of a cystic lymphangioma in the left cheek a few years before. On physical examination, in the right hypochondriac region, there is the presence of swelling of tense-elastic consistency, mobile on superficial and deep plans and painless, about 10 cm in maximum diameter. Ultrasound, CT and MRI documented the presence of an oval formation, with cystic charateristics 144x68 mm in diameter that extends caudally to about 10 mm in the context of the peritoneum in close proximity with the front lower edge of the small wing of the liver and the gastric body. The patient underwent exploratory laparotomy, where we found a massive lesion, well encapsulated, of elastic consistency, brown stalk, originating from the hepato-gastric ligament and we proceed with the removal of the mass “en bloc” after ligation of the vascular pedicle. No additional excision was necessary. The histological examination demonstrated a cystic lymphangioma. The post-operative course was regular, the patient was discharged on the third day. After six months of follow up the patient is disease free.

## Results

The cystic lymphangioma is a rare, hamartomatous tumor whose etiopathogenesis seems secondary to a congenital malformation of the lymphatic system [3,4]. The incidence is extremely low, showing a peak in childhood [4]. The most common site is in the superficial subcutaneous tissue in the neck, cheeks and supraclavicular region, and is rarely found at a deep level in the axillary region, mediastinal or abdominal [5]. In relation to the rarity, the diagnosis of LC may be a suspicion and arises after excluding other diseases with similar findings [6,7]. The complete surgical excision is the treatment of choice. The use of sclerosing injections may be indicated in unresectable lymphangiomas [8].The complete resection is without risk of recidivism, this possibility increases with incomplete removal, 10-15% with a tendency to invasive growth [9,10].

## Conclusions

The LC is a rare disease in adults; the authors describe the case of a person 71 years of age with a cystic lymphangioma of the peritoneum of the lesser omentum. Ultrasound, CT and magnetic resonance imaging for evaluating the characteristics and location of the tumor. The surgical excision is the treatment of choice, allowing a histological diagnosis.
